# Patient-reported outcomes and home-based self-swabs for influenza-like illness events - lessons learned from the 2023/2024 DANFLU-2 Homeswab PRO substudy

**DOI:** 10.1186/s41687-025-00936-8

**Published:** 2025-08-22

**Authors:** Filip Soeskov Davidovski, Kristoffer Grundtvig Skaarup, Niklas Dyrby Johansen, Daniel Modin, Nabila Shaikh, Jose Bartelt-Hofer, Matthew M. Loiacono, Rebecca C. Harris, Carsten Schade Larsen, Lykke Larsen, Lothar Wiese, Michael Dalager-Pedersen, Randi Jessen, Nina Steenhard, Brian L. Claggett, Scott D. Solomon, Lars Køber, Pradeesh Sivapalan, Jens Ulrik Staehr Jensen, Cyril Jean-Marie Martel, Tor Biering-Sørensen

**Affiliations:** 1 Cardiovascular Non-Invasive Imaging Research Laboratory, Department of Cardiology, Copenhagen University Hospital - Herlev and Gentofte, Copenhagen, Denmark; 2 Center for Translational Cardiology and Pragmatic Randomized Trials, Department of Biomedical Sciences, Faculty of Health and Medical Sciences, University of Copenhagen, Copenhagen, Denmark; 3 Sanofi, Reading, UK; 4 Sanofi, Lyon, France; 5 Sanofi, Swiftwater, PA USA; 6 Sanofi, Singapore; 7 Department of Clinical Medicine–Department of Infectious Diseases, Aarhus University Hospital, Aarhus, Denmark; 8 Department of Infectious Diseases, Odense University Hospital, Odense, Denmark; 9 Department of Infectious Diseases, Zealand University Hospital, Roskilde, Denmark; 10 Department of Infectious Diseases, Aalborg University Hospital, Aalborg, Denmark; 11 Department of Clinical Medicine, Aalborg University Hospital, Aalborg, Denmark; 12 Department of Virology and Microbiological Preparedness, Statens Serum Institut, Copenhagen, Denmark; 13 Cardiovascular Division, Brigham and Women’s Hospital, Harvard Medical School, Boston, MA USA; 14 Department of Clinical Medicine, Faculty of Health and Medical Sciences, University of Copenhagen, Copenhagen, Denmark; 15 Department of Cardiology, Copenhagen University Hospital – Rigshospitalet, Copenhagen, Denmark; 16 Respiratory Medicine Section, Department of Medicine, Copenhagen University Hospital – Herlev and Gentofte, Copenhagen, Denmark; 17 Steno Diabetes Center Copenhagen, Copenhagen, Denmark

**Keywords:** Self-swabs, Digital patient-reported outcomes (PRO), Influenza-like illness (ILI), Home-based testing, Feasibility study, DANFLU-2 trial, Compliance, Respiratory intensity and impact questionnaire (RiiQ)

## Abstract

**Background:**

Self-swabs and digital patient-reported outcomes (PROs) offer innovative tools for decentralized monitoring of infectious diseases. The DANFLU-2 HomeSwab PRO substudy evaluated the feasibility of using these methods for tracking influenza-like illness (ILI) within a large-scale, pragmatic, randomized trial.

**Methods:**

During the 2023/2024 influenza season, adults aged ≥ 65 years were recruited from the DANFLU-2 trial, which evaluates the relative effectiveness of high-dose influenza vaccine compared to standard-dose. Participants were instructed to self-swab at home upon ILI symptom onset and complete the Respiratory Infection Intensity and Impact Questionnaire (RiiQ™) for 14 days. Swabs were registered via QR code in a webapp and mailed for centralized PCR testing. Compliance was defined as completing all 14 days of RiiQ™ reporting.

**Results:**

Among 1,976 enrolled participants, 208 (10.5%) completed at least one RiiQ™, and 171 (82.2%) met the ILI case definition. Most participants found self-swabbing easy (66.1%) and more practical than visiting a clinic (78.6%). Compliance with daily RiiQ™ symptom tracking was 85.7%. Among those with ILI, 89.4% performed a self-swab within 1 day [IQR: 0; 3] of symptom onset; 65.8% of swabs were correctly registered in the webapp, and 96.5% were RNaseP-positive. Thirty-six participants (1.8%) withdrew, mainly due to weekly reminders; allowing reduced reminder frequency improved retention.

**Conclusion:**

The study confirmed the feasibility of using home-based self-swabs for remote disease diagnosis and digital PRO tracking for symptoms during ILI events in a large-scale, pragmatic randomized trial. While the approach proved viable, the findings also highlighted areas for improvement in participant engagement and data collection efficiency.

**Clinicaltrials.gov ID:**

NCT05517174.

**Supplementary Information:**

The online version contains supplementary material available at 10.1186/s41687-025-00936-8.

**Key Lessons Learned**:

1. **Swab Registration Improvements**: The relatively low success rate of correct swab registrations underscored the need for simpler workflows. For the 2024/2025 season, swabs are pre-linked to participant IDs, eliminating manual errors and reducing participant burden.
2. **Reduced Reminder Frequency**: The weekly reminder system, while effective, contributed to participant dropouts. The adoption of a less frequent and more flexible reminder schedule is expected to improve retention without compromising compliance.
3. **Shortened Follow-Up Period**: The 14-day RiiQ period was excessive for some participants. Transitioning to a 7-day follow-up, with extensions to 14-days for participants with persistent symptoms, reflects a balance between data quality and participant convenience.

## Introduction

Seasonal infectious diseases remain a significant global public health burden, particularly among older adults, who are at increased risk of severe outcomes [1]. Traditional surveillance systems, which rely on healthcare visits and laboratory testing, often underestimate community-level disease burden, as many individuals manage their symptoms at home [2, 3]. This discrepancy highlights the need for innovative approaches to monitor influenza-like illness (ILI) and other respiratory illnesses [4, 5], particularly in high-risk populations such as those aged 65 years and older [1]. Decentralized and patient-centered methodologies have emerged as transformative tools in clinical research, enabling real-time, scalable data collection while reducing participant burden and geographical barriers [6]. These approaches proved valuable during COVID-19, enabling effective self-swabbing and patient-reported outcomes (PRO) data collection in community cohorts [4, 7, 8]. Importantly, regulatory agencies such as the U.S. Food and Drug Administration (FDA) and the European Medicines Agency (EMA) are increasing their focus on laboratory confirmation of cases in vaccine efficacy trials and using this as an endpoint for approving new vaccines [9–11]. This new focus underscores the critical role of home-based self-swabbing with laboratory confirmation, as it would be able to generate robust, laboratory-confirmed data in randomized studies without patients coming to a clinic for testing.

 [12]. Such an approach has created considerable interest from the vaccine development community, which views home swabbing as a promising method to deliver patient-centric vaccine trials. In addition, patient-reported outcomes (PROs), in particular, offer unique insights into disease impact and treatment outcomes from the patient’s perspective [10, 11]. However, limited research exists on the feasibility of using remote methods for home self-collection of swabs and shipment for centralized PCR analysis, as well as digital PRO reporting of ILI symptoms among older adults. This group may face mobility and digital barriers affecting swab use and symptom reporting [13]. Despite these challenges, older adults may benefit most from patient-centric designs that simplify respiratory vaccine trial participation.

The 2023/2024 DANFLU-2 HomeSwab PRO substudy was designed to evaluate the feasibility of decentralized ILI monitoring using self-administered nasal and oropharyngeal swabs combined with digital symptom tracking via the Respiratory Intensity and Impact Questionnaire (RiiQ™) [14] among older participants in a large-scale pragmatic individually randomized trial. Conducted as part of the broader DANFLU-2 randomized controlled trial, which is an ongoing pragmatic randomized trial evaluating the effectiveness of high-dose influenza vaccine vs. standard-dose influenza vaccine in older adults, the population included individuals aged ≥ 65 years, reflecting the demographic most at risk for complications from seasonal respiratory infections, hence ILI [1, 15]. By leveraging digital tools and home-based sampling, the study aimed to overcome barriers to participation, increase engagement, and provide robust data on symptom burden and virological outcomes [6].

This paper discusses the key operational and feasibility-related findings from the 2023/2024 DANFLU-2 HomeSwab PRO substudy, the challenges encountered, and the adjustments made to optimize the protocol for the subsequent season, which will include a substantially larger sample. It also contributes to future related studies that seek to leverage the advantages of decentralization.

## Methods

The DANFLU-2 HomeSwab PRO substudy was conducted during the 2023/2024 influenza season as a sub-study of the DANFLU-2 randomized controlled trial (ClinicalTrials.gov: NCT05517174). Participants aged 65 years or older already enrolled in the DANFLU-2 trial were recruited prior to randomization from various vaccination sites across the Capital Region of Denmark, between September 28^th,^ 2023, and October 2^nd,^ 2023 (flowchart in Table 1). Eligible participants were asked to participate in the HomeSwab PRO substudy before being randomized 1:1 to receive either high-dose or standard-dose influenza vaccine in the DANFLU-2 trial. Upon enrollment in the HomeSwab PRO substudy, participants were instructed to self-monitor symptoms of ILI, if occurring, from two weeks after vaccination until the end of follow-up (May 31, 2024). An ILI event was defined using the the European Centre for Disease Prevention and Control (ECDC) case definition as follows: Sudden onset AND at least one among: fever (body temperature above or equal to 38 °C), feverishness, headache, malaise, myalgia AND at least one among: cough, sore throat, shortness of breath.

**Table 1:**
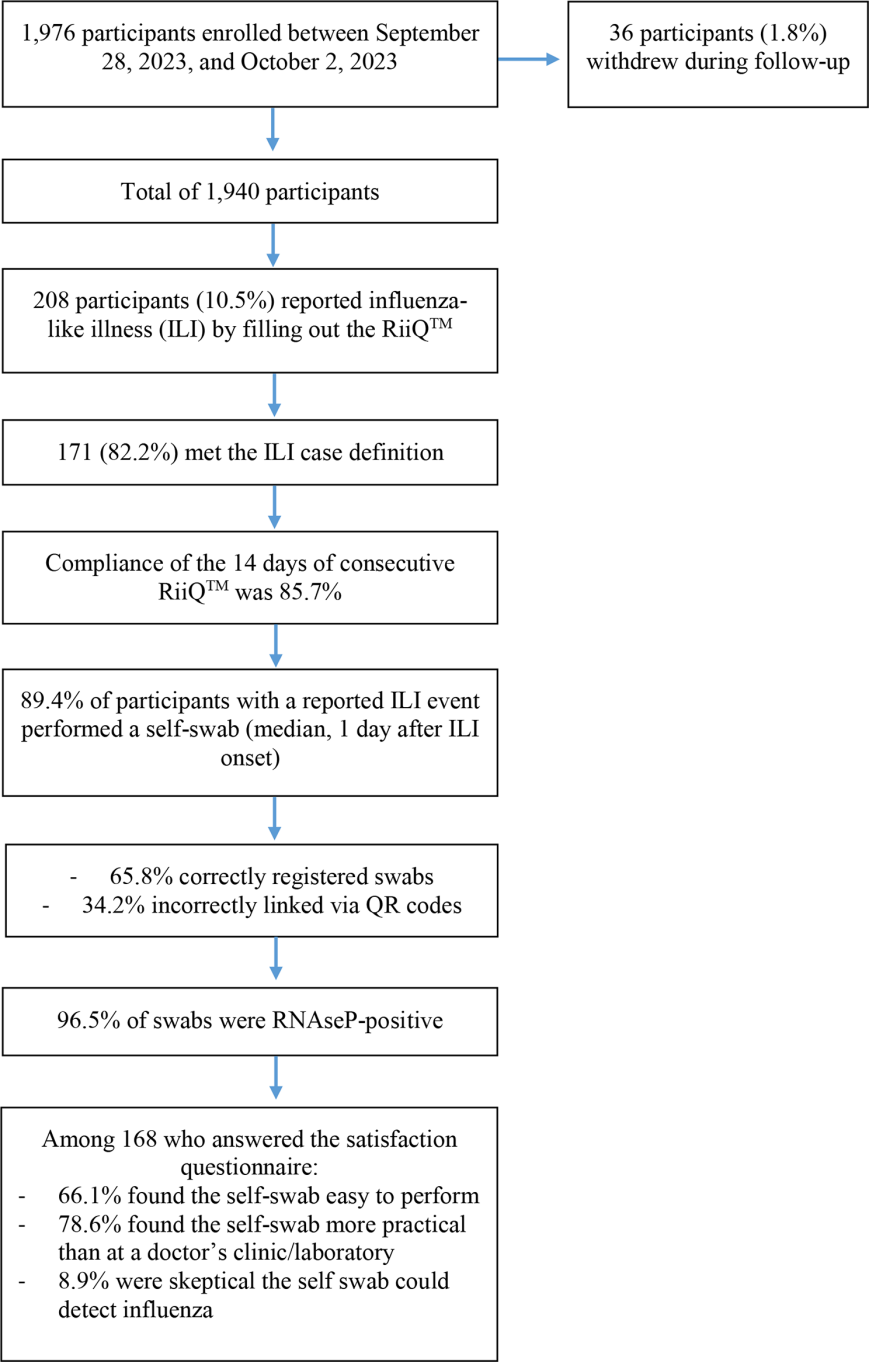
Flowchart of participant enrollment, dropouts, ILI events, Self-Swabbing, and RiiQ™ completion

**Fig. 1:**
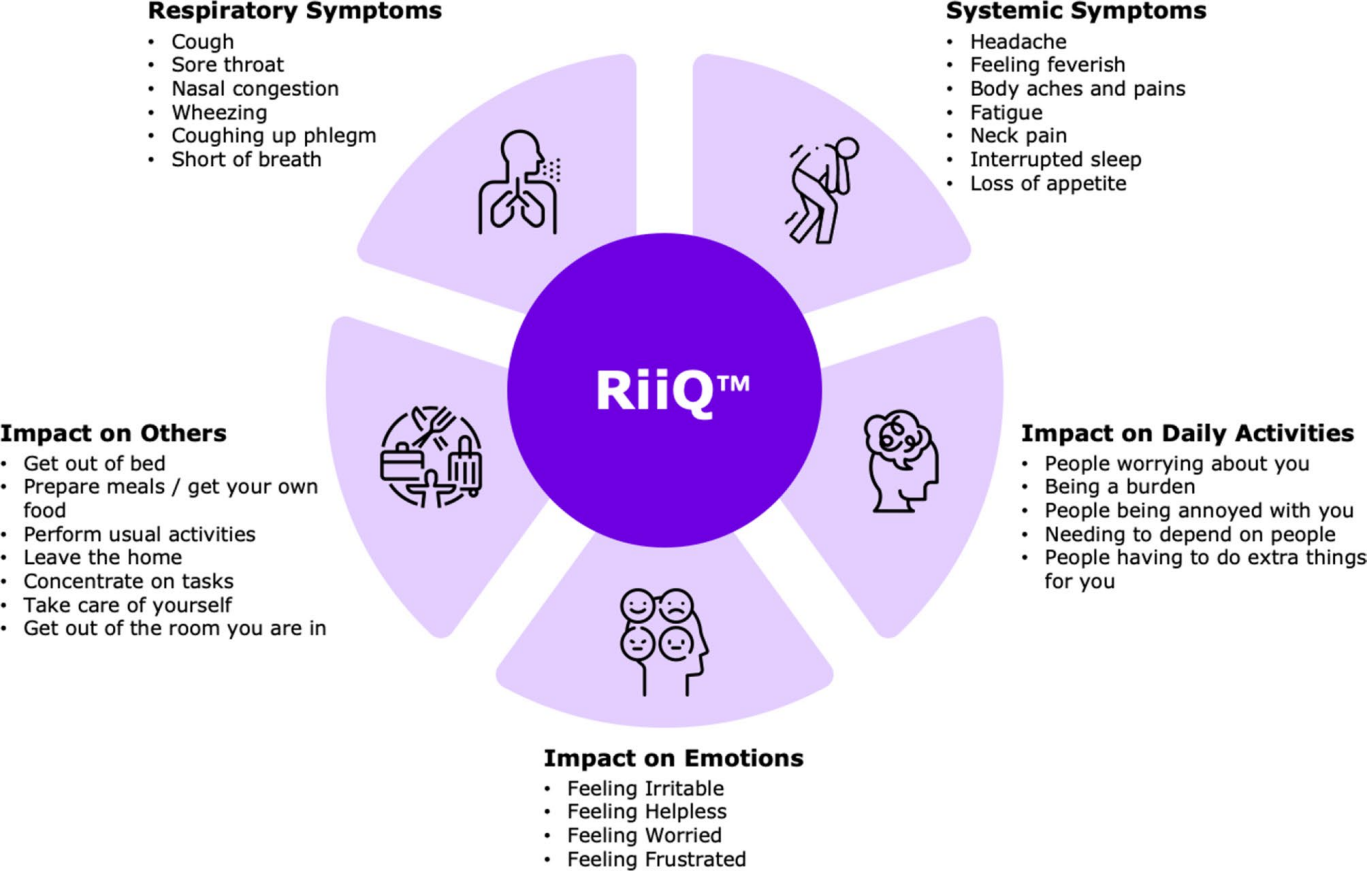
Overview of RiiQ™ Domains

Participants experiencing ILI symptoms were prompted to begin daily reporting using the RiiQ™ questionnaire, a disease-specific PRO instrument for respiratory diseases [14]. The RiiQ™ is an extension of the Influenza Intensity and Impact Questionnaire (FluiiQ™), a widely used PRO for respiratory infection studies [16], which was refined to measure a wider range of respiratory diseases [16], including influenza and Respiratory Syncytial Virus (RSV), among others [14, 17]. The RiiQ™ comprises five domains (Fig. 1): (a) respiratory symptoms, encompassing six upper and lower respiratory tract symptoms (e.g., cough, sore throat); (b) systemic symptoms, including seven systemic manifestations (e.g., fever, fatigue); (c) impact on daily activities, assessed through seven items; (d) impact on emotions, measured with four items; and (e) impact on others, evaluated through five items. Each symptom and impact is rated on a 4-point scale (e.g., 0 = none; 1 = mild; 2 = moderate; 3 = severe), with higher scores denoting greater severity or impact. Participants were instructed to digitally complete the RiiQ™ questionnaire daily for 14 days following symptom onset, with a maximum recall period of 24 h, thereby accurately capturing changes in symptom severity, and functional impact throughout the course of the illness.

**Fig. 2:**
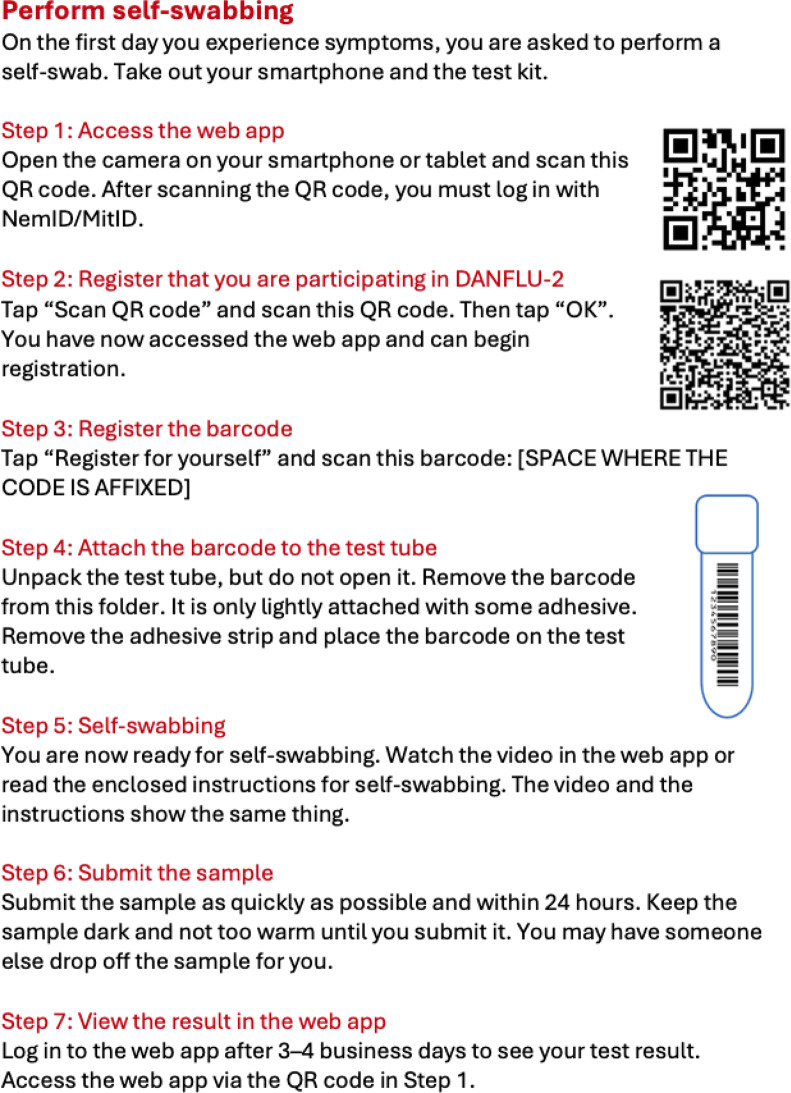
Illustration of QR-code instruction for participants

Participants received standardized self-swab kits during enrollment and before randomization, which included a sample tube containing Universal Transport Media (UTM), a FLOQSwab for nasal and oropharyngeal sampling, along with detailed written instructions for sample collection and packaging. Symptomatic participants could register a sample by scanning a QR code to enter the custom-designed smartphone web application, and then a unique sample barcode, thereby linking the sample to the participant. Each kit included QR and sample codes to link test results to participant data (Fig. 2). Participants were instructed to perform self-swabbing as soon as possible after symptom onset and mail the samples in prepaid envelopes to a central laboratory at Statens Serum Institute for analysis. To encourage adherence, participants received automated weekly reminders via Denmark’s secure mandatory governmental communication platform, Digital Post/eBoks, which enables secure digital communication with citizens. Weekly reminders (Supplemental Fig. 1) included ILI criteria, mailing location, and RiiQ™ link; daily reminders (Supplemental Fig. 2) followed symptom onset. For participants who failed to respond to two consecutive RiiQ™ questionnaires (not reporting symptoms for 48 h), follow-up calls was done to support reporting and swab completion. After completing the 14-day RiiQ™, participants assessed ease of swabbing, preference vs. clinic testing, and confidence in detecting influenza. Data on compliance, sample quality, and participant retention were monitored throughout the trial to assess areas of potential improvement in study processes. The team also addressed participant e-mail queries about logistics, swab registration, and questionnaire use.

## Results

In the 2023/2024 influenza season, the DANFLU-2 HomeSwab PRO substudy enrolled 1,976 participants. During the study period, 208 participants (10.5%) reported ILI symptoms, among them 171 (82.2%) met the study’s ILI case definition. Compliance with the RiiQ™ questionnaire was 85.7% for completing all 14 days of RiiQs among the symptomatic participants. In total, 107 (51.4%) of the 208 required phone reminders due to missing two consecutive RiiQs (a 48-hour non-response period from reporting their symptoms) within the collection period, with 91 (85%) requiring 1 contact attempt per non-responding participant. While RiiQ™ was generally user-friendly, 44 (26.2%) of 168 found the 14-day period excessive, especially in case of mild symptoms. Among respondents, 111 (66.1%) found self-swabbing easy, and 132 (78.6%) preferred it over clinic testing, while 15 (8.9%) expressed doubt regarding the ability of the self-swab to reliably detect influenza. Swab compliance was similarly robust, with 89.4% of symptomatic participants completing a RiiQ™ questionnaire performing a self-swab; performed within a median of 1 day [IQR: 0; 3] after symptom onset. 34.2% of all samples were not registered correctly in the webapp, and thereby, it was not possible to link the swab and the test results to the participant´s study ID. Swab-sample quality was high, with 96.5% of swabs testing positive for RNAseP, indicating that most participants followed the collection instructions correctly.

Retention throughout the trial was also high, with 36 participants (1.8%) withdrawing from the substudy. Withdrawals (median: 59 days after enrollment) were mainly due to reminder burden or critical health deterioration. To reduce dropouts, the possibility of adjusting the frequency of reminders was offered, with 40 participants (2.0%) subsequently opting to receive monthly reminders and 47 (2.4%) opting to receive no further reminders during the study period. The team handled 323 e-mail queries throughout the study, mainly about swab registration, questionnaires, or reminders.

## Discussion

The 2023/2024 DANFLU-2 HomeSwab PRO substudy demonstrated that decentralized methodologies, including self-swabs and digital PROs and the broader feasibility of remote disease diagnosis and digital symptom tracking, are feasible and effective for monitoring ILI in a s large-scale pragmatic randomized trial setting. The high compliance rates for swab collection and symptom reporting emphasize the willingness of older adults to engage in home-based research despite unique challenges. While 10.5% of participants reported ILI symptoms during follow-up, this proportion is consistent with seasonal ILI incidence rates of 7–11% observed in prior prospective studies among older adult [18–20]. However, as with other studies based on self-reported symptoms, we cannot exclude the possibility that some participants experienced ILI but did not report it - an inherent limitation of this decentralized study design. A more active surveillance approach, such as mandatory weekly check-ins, could potentially reduce any underreporting, but would also increase participant burden and could compromise retention in this population.

Our cohort (≥ 65 years) was older than those in prior studies like TestBoston (mean age 47 years old) and Seattle Flu Study (80.6% between 25 and 64 years of age) [7, 21]. The older age group in our trial presented additional challenges, including possible lower IT literacy compared to younger people, contributing to difficulties registering self-swabs in the webapp, through a QR code, linking the swab to the participant ID (only 65.8% success). This is despite Denmark’s high digital infrastructure and leading internet use among older adults in Europe [22]. The findings of digital barriers are emphasized in prior studies, such as Money et al. and Heponiemi et al., which highlighted logistical and digital barriers in older individuals’ participation in online health services compared to younger people [23, 24]. To address this, our protocol for the subsequent 2024/2025 season of the DANFLU-2 HomeSwab PRO substudy includes pre-linking of swabs to participant IDs before shipping the unique labelled test kit to the participant’s home address instead of handing them out at the vaccine site, thereby simplifying the swab registration process for the participant. Despite these barriers, the trial achieved a retention rate of 98.2%, with only 36 participants withdrawing to participate, demonstrating the value of tailored support measures, such as adjustable reminders and phone follow-ups for non-responders.

The high quality of swab samples (96.5% RNAseP-positive) aligns with findings from Goyal et al., who reported that 92% of received self-swabs were considered adequate samples in a cohort of adults ≥ 65 years in Thailand [13]. However, the 14-day duration of the RiiQ proved burdensome for some participants, particularly those with milder symptoms, similar to Gander et al., who found daily survey completion dropped as symptoms resolved [25]. In response, the reporting period has been reduced to 7 days in the 2024/2025 season, with an extension to 14 days only if symptoms persist at day 7.

Importantly, our decentralized design, which incorporates home-based self-swabbing, addresses a new critical regulatory focus for laboratory confirmation of cases in vaccine efficacy trials as an additional method rather than merely based on immunogenicity, mandated by agencies such as the FDA and EMA [9–11]. By facilitating the collection of robust, laboratory-confirmed data within a randomized trial framework among older adults, this approach not only enhances the efficacy assessments but also holds significant promise for future vaccine trials. The scalability and flexibility demonstrated in our trial suggest that decentralized methodologies can streamline trial logistics and improve regulatory compliance, thereby accelerating the approval process for new vaccines.

Overall, the 2023/2024 DANFLU-2 HomeSwab PRO substudy highlights the potential and challenges of decentralized research within respiratory infectious diseases in older adults. This adaptation laid the groundwork for the 2024/2025 season, which included > 10,000 participants as of October 2024.

## Conclusion

The 2023/2024 DANFLU-2 HomeSwab PRO substudy provided key insights into implementing decentralized methods for ILI monitoring in older adults within a large-scale randomized trial. The study demonstrated feasibility and high compliance among the responding participants with ILI, but also identified areas for improvement, particularly regarding IT swab registration, reminder systems, and the duration of the symptom reporting period. Lessons from this study can guide future decentralized self-swab and PRO research in respiratory diseases.

## Supplementary Information

Below is the link to the electronic supplementary material.

Supplementary Material 1

## Data Availability

The datasets used and/or analysed during the current study are available from the corresponding author on reasonable request.

## Abbreviations

ECDC: European Centre for Disease Prevention and Control
EMA: European Medicines Agency
FDA: U.S. Food and Drug Administration
ILI: Influenza-like illness
PRO: Patient-reported outcome(s)
REDCap: Research Electronic Data Capture Platform
RiiQ™: Respiratory Infection Intensity and Impact Questionnaire
RNAseP: Ribonuclease P
