# Supplementary material for: Patient-reported outcomes and home-based self-swabs for influenza-like illness events - lessons learned from the 2023/2024 DANFLU-2 Homeswab PRO substudy
Source: J Patient Rep Outcomes. 2025 Aug 22;9:108. doi: 10.1186/s41687-025-00936-8 (PMC12373587; doi:10.1186/s41687-025-00936-8)

**Supplemental Figure 1. Weekly reminder to participants through Digital Post/e-Boks**


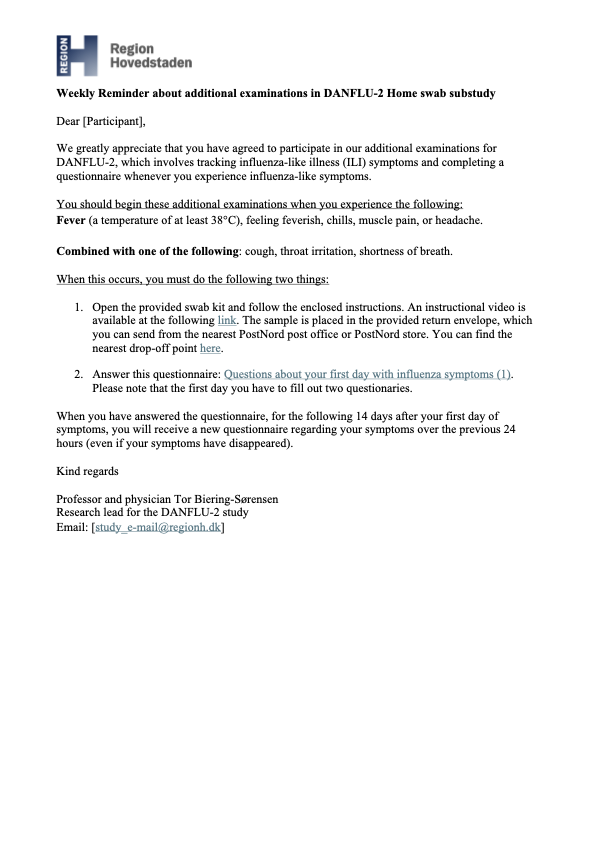


**Supplemental Figure 2. Daily reminder to report symptoms in the RiiQ questionnaire through Digital Post/e-Boks**


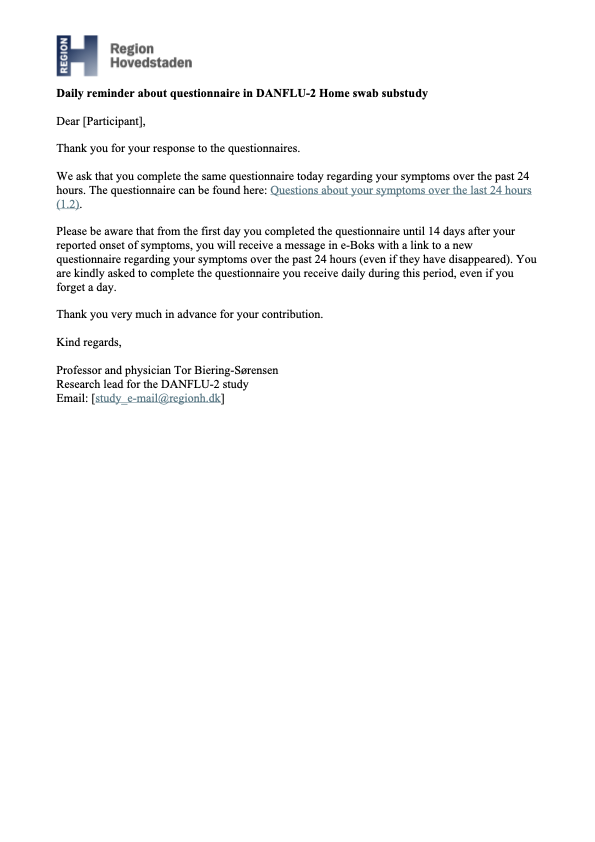

Supplement: Supplementary file 1 — Supplementary Material 1 [file 41687_2025_936_MOESM1_ESM.docx]
